# A landscape analysis of clinical trials and infant clinical trials in Kenya, Ethiopia, and Nigeria

**DOI:** 10.3389/fepid.2024.1417419

**Published:** 2024-08-15

**Authors:** Patrick Amboka, Daniel Kurui, Marylene Wamukoya, Julius Kirimi Sindi, Marta Vicente-Crespo

**Affiliations:** ^1^ Research and Related Capacity Strengthening Division, African Population and Health Research Center, APHRC, Nairobi, Kenya; ^2^ School of Public Health, University of the Witwatersrand, Johannesburg, South Africa

**Keywords:** clinical research, infant and intervention clinical trials, RCT, LMIC, randomized trials, existing capacity, capacity strengthening

## Abstract

**Introduction:**

Global inequality in clinical research capacity and service delivery can be indicated simply by the proportion of clinical trials that a country or region has registered in clinical trial registry databases. The proportion of clinical trials registered in Africa is very low at 0.02%, even though the region accounts for approximately 15% of the world's population. Despite the economic challenges in most African countries, they have shown potential for growth and change in recent years.

**Methods:**

We conducted desk reviews on the interventional clinical trials done in Kenya, Ethiopia, and Nigeria between 2015 to May 2023. The search was done in clinical trials repositories, and journal repositories. The search focused on intervention clinical trials. Data was extracted by screening through the publications and clinical trial platforms. The data extracted from the publications included the type of clinical trial, clinical trial phase, diseases, etc. The data extracted from the reports included: challenges in conducting clinical trials, capacity-building efforts, and the impact of the clinical trial.

**Results:**

The number of clinical trial studies identified in Kenya was 113 (28 were on infant clinical trials). The study identified 97 clinical trials in Nigeria, of which 11 studies were on infant clinical trials. In Ethiopia, there were 28 clinical trials and only five were on infant clinical trials. The landscape review also expanded to capacity and gaps in clinical trials in the three countries. The largest proportion of clinical trials carried out in Kenya was on injury, occupational disease, and poisoning, 30.5% (*n* = 18) and the smallest proportion was on kidney disease, neonatal disease, obstetrics, and gynecology. Most Infant clinical trials were carried out in the area of infections and infestations 33.3% (*n* = 7). Most of the challenges faced by clinical trials in the three countries include a lack of infrastructure, a lack of human resources, and a lack of financial resources.

**Implications:**

There is a need to map clinical trials done by African researchers based in Africa to exclude the trials done by non-African researchers based in Africa. Opportunities for clinical trials should be supported and challenges addressed.

## Introduction

Global inequality in clinical research capacity and service delivery can be determined by the proportion of clinical trials that a country or region has registered in clinical trial registry databases. According to the World Health Organization (WHO), clinical trials are a type of research that studies new tests and treatments and evaluates their effects on human health outcomes (1). Eighty-three percent of clinical trials have been conducted in 25 high-income countries and only 5% have been conducted in 91 low and middle-income countries (LMIC) (2). The proportion of clinical trials registered from Africa (on clinicaltrials.gov) is only 0.02%, even though the region accounts for around 15% of the world's population (3). In Sub-Saharan Africa (SSA), most clinical trials are conducted in South Africa (47.3%), which has one of the largest financial investments in health and a relatively well-developed healthcare system (4). Only a few studies in Kenya, Ethiopia, and Nigeria are registered on clinical trial registries and platforms. There are also substantial capacities and gaps in clinical trials in these particular countries.

Based on the low proportions of clinical trials registered in Africa, this landscape analysis focuses on the number of interventional clinical trials in Kenya, Ethiopia, and Nigeria and also the landscape of existing capacity and gaps in clinical trials in the three countries. Ethiopia, Nigeria, and Kenya are among the largest countries in Africa in terms of population (3). Therefore, they are expected to carry out more clinical trials but that is not the case. These clinical trials seek to find out more about an intervention or treatment while observational studies on the other hand seek to find out the consequences to people in different situations (1). Observational studies do not include intervention; therefore, they do not follow the clinical trial phases (5). WHO classifies intervention clinical trials into 4 phases: Phase I, Phase II, Phase III, and Phase IV (2). Phase 0 studies were not considered since they involve a very small number of participants receiving a very small dose of a drug and only help in speeding up and streamlining the drug approval process. Phase 0 studies only help researchers to find out what to do and this helps in saving time and resources that would otherwise be spent on later phases. In phase I, researchers test a drug/treatment on between 20 and 80 people for the first time to identify side effects and safety tests (6). In Phase II, the new drug/treatment is tested on a larger group of people (between 100 and 300 people) for effectiveness and further safety (6). In Phase III, the new drug/treatment is tested on a large group of people (between 1,000 and 3,000 people) for effectiveness, side effects monitoring, and comparisons with other similar treatments (6). In Phase IV, the drug/treatment is already approved by the Food and Drug Administration (FDA) and the public has access to it, the drug safety is tracked in the general population (6).

Despite the economic challenges, the three countries (Kenya, Ethiopia, and Nigeria) have shown potential for growth and change in recent years. For example, Ethiopia is the second-largest country in the world when it comes to the increase in life expectancy over the last two decades. Ethiopia has also made significant progress in reducing child mortality and maternal mortality and increasing preventative primary health care as well as in the number of medical schools (7).

In Kenya, Ethiopia, Nigeria, and Africa at large clinical trials are mostly carried out in institutions such as hospitals. This is because these hospitals provide ready participants for the clinical trials. However, there are a lot of challenges which include inadequate infrastructure at these particular hospitals that enables them to carry out clinical trial studies efficiently and quickly (4). The lack of dedicated research teams in these institutions, unreliable internet access, and lack of skilled researchers greatly affect the quality of clinical trials in these institutions (8).

There are also challenges about the weight being placed on clinical trials in specific thematic areas of infectious diseases, malnutrition, non-communicable diseases such as hypertension, and diabetes, genetic problems such as sickle cell disease, and culture despite the areas accounting for a large burden of diseases in Africa (9). SSA accounts for up to 50% of the global burden of disease, primarily due to infection (10). Clinical trials testing drugs and vaccines that target specific diseases affecting people in SSA are logically best conducted in these countries. Another challenge arises from partnerships and collaboration. The partnerships and collaborations among stakeholders in the clinical trial ecosystem reduce the burden on sponsors seeking regulatory jurisdiction in most countries and could make Africa competitive as a destination for clinical trials (11). Collaboration/partnership among national regulatory agencies will reduce administrative burden as well as enable Africa to be treated as one regulatory jurisdiction (11). Researchers can partner to attain necessary certification, improve quality, and increase overall efficiency and hence make clinical trial projects seamless in Africa (11). Clinical trials carried out in Africa often face other challenges which include limited financial and human capacity, delays in regulatory and ethical reviews, complex financial systems and logistics, and competing demands (12).

Clinical research in LMIC builds both research and health capacity which has been shown to strengthen health systems, expand health programs, and provide an evidence base for future responses to health crises (12, 13). Studies have shown that the key operational advantages of conducting studies in Africa include the ease of recruitment of study participants (12). Clinical trials are needed to find new and effective ways to diagnose and treat these diseases. Previous studies have shown that clinical trial studies can benefit local health knowledge as well as lead to more effective interventions (12). Clinical trials that are targeted at testing drugs and vaccines on specific diseases in SSA are logically best conducted in those specific countries (14). This landscaping provides invaluable insights into the current state of general interventional clinical trials and also interventional infant clinical trials in the three countries and helps to identify opportunities for improvement. By addressing the challenges and building on the opportunities, interventional clinical research in these countries can help inform policy and practice and contribute to the global body of knowledge on clinical research. Ultimately, patients from these African countries will benefit as the quality of clinical practice improves and access to new therapies is enhanced.

## Methodology

### Scoping review methodology

#### Sources of information

Information on clinical trials was obtained from the following sources:

- **Published literature**: A comprehensive review of published literature on interventional clinical trials conducted in these countries was conducted. This included peer-reviewed journal articles.
- **Clinical trial registries:** Clinical trial registries were reviewed, such as the Pan African Clinical Trials Registry (PACTR) (https://pactr.samrc.ac.za/) and Clinical Trials.gov (https://clinicaltrials.gov/) in order to identify ongoing and completed clinical trials in these countries.

#### Search criteria

(a) *Identification of relevant data sources*

Relevant data sources were identified, and they included:

- PubMed (https://pubmed.ncbi.nlm.nih.gov/)
- Clinical trial registries, such as the PACTR and Clinical Trials.gov (https://clinicaltrials.gov/). Information on institutions conducting clinical trials was extracted from the two registries by customizing the search to focus on the three countries.
- Other relevant sources of information, such as websites of regulatory agencies (NACOSTI) and professional organizations
- Grey literature was also searched on institutions' websites. A list of institutions conducting health research in the three countries was identified and information on clinical trials was extracted from their websites.

This initial search was then followed by an analysis of the text words contained in the title and abstract of retrieved papers, and of the index terms (subject headings) used to describe the articles. A second “systematic search” was then undertaken across all included databases and this search used all identified keywords and index terms. The reference list of identified reports and articles was searched for additional studies. This stage examined the reference lists of all identified studies and examined solely those studies that have been selected from full-text and/or included in the review. The search strategy followed the Population (or participants)/Concept/Context (PCC) framework which is recommended as a guide to constructing clear and meaningful objectives and eligibility criteria for a scoping review (15).

(b) *Development of search terms*

Search terms were developed so as to capture the relevant concepts related to clinical trials in the three countries. The initial search was done in MEDLINE/PubMed. Both keywords and medical subject headings (MeSH) were used. For each PCC element, the relevant keywords (MeSH) were created, and each line was given its own MeSH. The keyword lines were then entered and later joined to create a total set line for this PCC element by combining them with the phrase “OR”. Repetition was done for each PCC element. Thereafter, the total sentence lines were connected with the phrase “AND”. This helped in finding the results that took all of our PCC elements into account.

The search was conducted in the English language from 2015 up to May 2023. It used the following keywords: (Clinical Trials* AND (Diagnosis/Prognosis OR Early detection/Screening OR Other Interventions OR Physical activity and nutrition OR Prevention OR Prevention: Vaccines OR Psychosocial OR Rehabilitation OR Supportive care OR Treatment: Devices OR Treatment: Drugs OR Treatment: Other OR Treatment: Surgery) AND (Phase 0 OR Phase 1 OR Phase 2 OR Phase 3 OR Phase 4) AND countries of recruitment (Kenya OR Ethiopia OR Nigeria) AND principal investigator country (Kenya OR Ethiopia OR Nigeria).

To further narrow it down to only infant clinical trials (Clinical trials on children less than 24 months. However, some studies overlapped from newborn (0 day–30 days, through infants (1 month to 24 months) to preschool (24 months to 59 months). The search was also done in English using the following keywords: (Infant OR Newborn OR Child, OR Neonatal). All studies were read through carefully to ensure we only had clinical trials for infants. After the MEDLINE search, the search criteria were run on other databases and were optimized as necessary for each database.

(c) *Screening*

After the search strategy was created, the final version was saved to each database identified. We performed our search on each database and exported all the results to EndNote. In EndNote, we used a separate group to store the results from each database. We kept our EndNote library safe and secure and later relied on it for full text. The references were exported from EndNote and imported into Covidence (https://www.covidence.org/) for screening. Covidence software made it possible to retain relevant data and identify duplicates that were later deleted. Since the studies were screened by two authors, automating the screening and identification of duplicates was key to removing the double entry.

#### Inclusion and exclusion criteria

For each individual search term, we considered the first three pages of the results (16). For all searches, we looked at the header and summary of results. A clinical trial was included in the original list if:

1. The title contained relevant terms such as “clinical trials” and ‘randomized clinical trials.
2. It had not been included in a previous search or recommendation. We did not exclude clinical trials based on the researcher's country of origin but rather inclusion focused on where the researcher was currently based.

Conversely, a clinical trial was excluded if:

1. Observational studies which did not require the four phases of clinical trials were excluded.
2. Due to time and resource constraints, we limited our search to English-language resources.
3. Phase 0 studies were deemed not feasible by respective researchers after involving a very small number of participants receiving a very small dose of a drug.
4. Phase 0 studies only help researcher researchers to find out what to do and this helps in saving time and resources.

#### Data extraction

We developed a standardized data extraction tool designed to capture details about the clinical trials, that is, thematic area, institution, study site, the phase of clinical trials, collaborating institutions, timeframe, country, recruitment status, publication URL, disease, age group, gender, recruitment center name, contact name, contact email, contact city, and contact position. Data extraction was performed by the first author and subsequently checked for accuracy by other authors. Entities were eligible for full extraction if the clinical trial met the following criteria:

1. Met the definition of an interventional clinical trial (17)
2. Focused on phases (I, II, III, and IV). Phase 0 studies were excluded.
3. Conducted for the last 8 years at the time of review (from 2015 to May 2023)
4. The description and relevant materials were in English.

Data elements relevant to the review questions were extracted and analysis was performed (typically via frequency counts and/or basic qualitative content analysis). Data were presented using tables, visualizations, and narrative summaries to achieve the objectives of the scoping review and the landscape analysis and to answer the review questions. The missing data on the expected start date and completion date of clinical trials was set to “1/1/1900” to show that the data on the dates was missing.

#### Synthesis of the findings

The findings were synthesized from the data analysis to develop a comprehensive assessment of the current state of interventional clinical trials in Kenya, Ethiopia, and Nigeria, and to identify opportunities for improving the quality and quantity of clinical research in these countries. The first set of data was for general interventional clinical trials and then the search narrowed down to interventional infant clinical trials in Kenya, Ethiopia, and Nigeria.

## Project data on clinical trials

### Data extraction

The authors reviewed the project's grey literature reports on clinical trials to extract relevant data on the clinical trials. The project's reports included experiences of researchers working/who have worked on interventional clinical trials or whose focus included interventional infant clinical trials. These data were in addition to the scoping review data that had been conducted previously. The data extracted from the project's reports included clinical trial thematic areas, a list of collaborating partners, challenges in conducting the clinical trials, their capacities in conducting clinical trials, opportunities for joint research projects, their capacity-building initiatives, the impact of the clinical trial, and areas for potential collaboration. Data on the successes and lessons learned from the clinical trials was also extracted. Data extraction were done until the thematic saturation was reached (*n* = 28) (18). The thematic grouping was harmonized by the author based on the registries grouping to only capture themes of interest/relevant themes. The documents that were used in the data extraction were from the project data not published.

### Analysis

The data extracted were analyzed using thematic content analysis (18) with prior themes forming the basis for the analysis and focusing on the challenges of conducting clinical trials, institutional capacities to conduct clinical trials, opportunities for joint collaboration/capacity-building initiatives, and areas for potential collaboration. These results were combined with data extracted from institutions' websites, publications, and clinical trial platforms.

## Results

### Scoping review

#### Selection of sources of evidence

**Figure 1:**
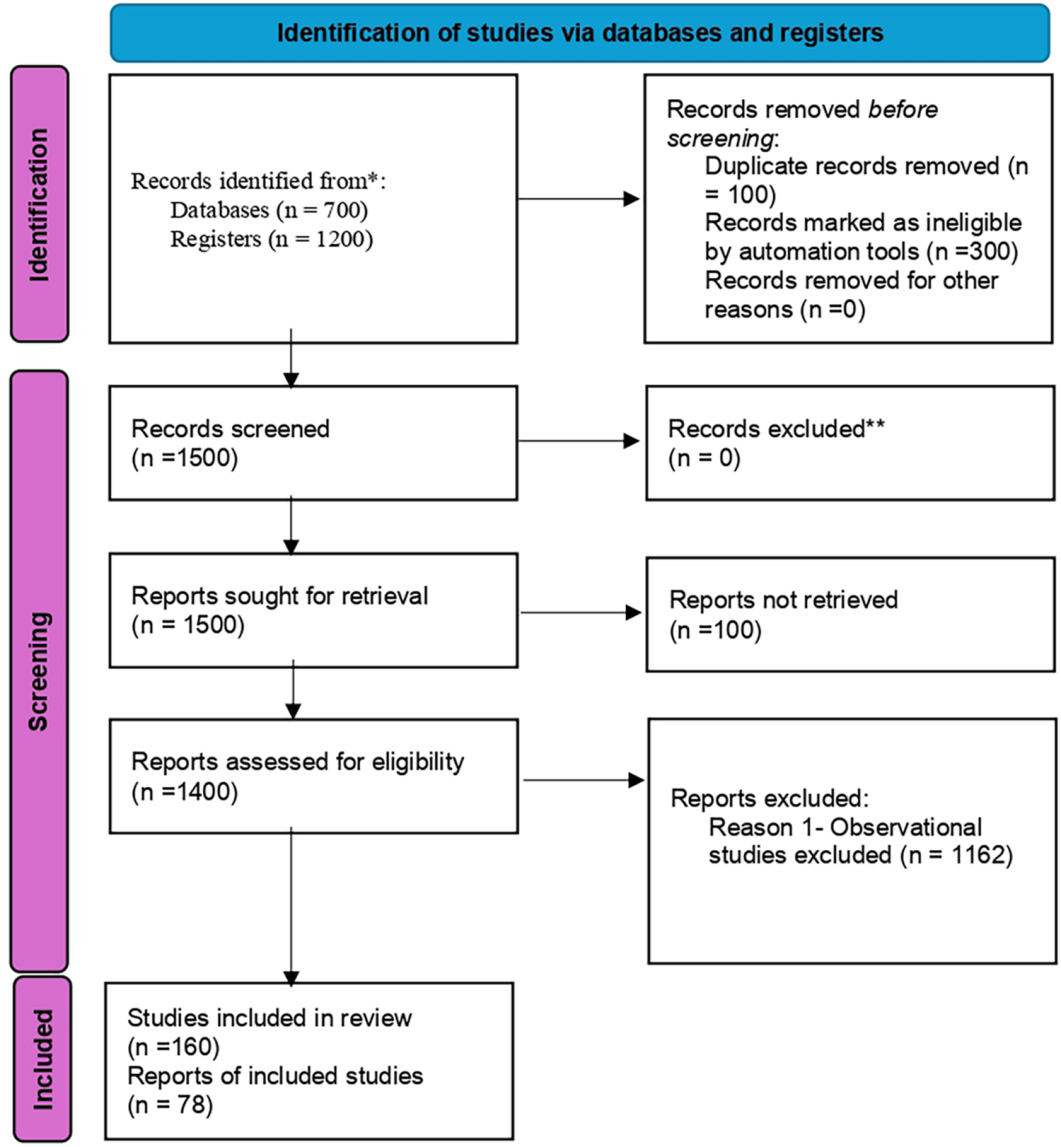
The PRISMA 2020 flow chart for identification of studies via databases and registers.

Figure 1 shows the identification of studies via databases and registers.

The landscaping exercise identified 24 institutions in Ethiopia carrying out interventional clinical trials: 28 interventional clinical trial studies, and 5 interventional infant clinical trial studies. For more details, (see the Supplementary File: Clinical trials data for Ethiopia).

In Kenya, the landscaping identified 17 institutions carrying out interventional clinical trials: 113 studies on interventional clinical trials, and 28 studies on interventional infant clinical trials (see Supplementary File: Clinical trials data for Kenya).

In Nigeria, the landscaping identified 32 institutions carrying out interventional clinical trials: 97 studies on interventional clinical trials, and 11 studies on interventional infant clinical trials (see Supplementary File: Clinical trials data for Nigeria). These findings are presented in Table 1.

**Table 1:** Summary of clinical trials and infant clinical trials in Ethiopia, Kenya, and Nigeria.

Country	Number of institutions carrying out clinical trials	Number of studies on clinical trials	Phase I clinical trial studies	Phase II clinical trial studies	Phase III clinical trial studies	Phase IV clinical trial studies
Ethiopia	24	28	3	1	5	9
Kenya	17	113	17	34	42	20
Nigeria	32	97	12	24	44	17
	Institutions carrying out infant clinical trials	Number of infant studies on infant clinical trials	Phase I clinical trial studies	Phase II clinical trial studies	Phase III clinical trial studies	Phase IV clinical trial studies
Ethiopia	3	5	1	1	2	1
Kenya	7	28	5	8	11	4
Nigeria	16	11	0	2	4	5

#### Interventional clinical trials in Kenya, Ethiopia, and Nigeria by thematic area

**Figure 2:**
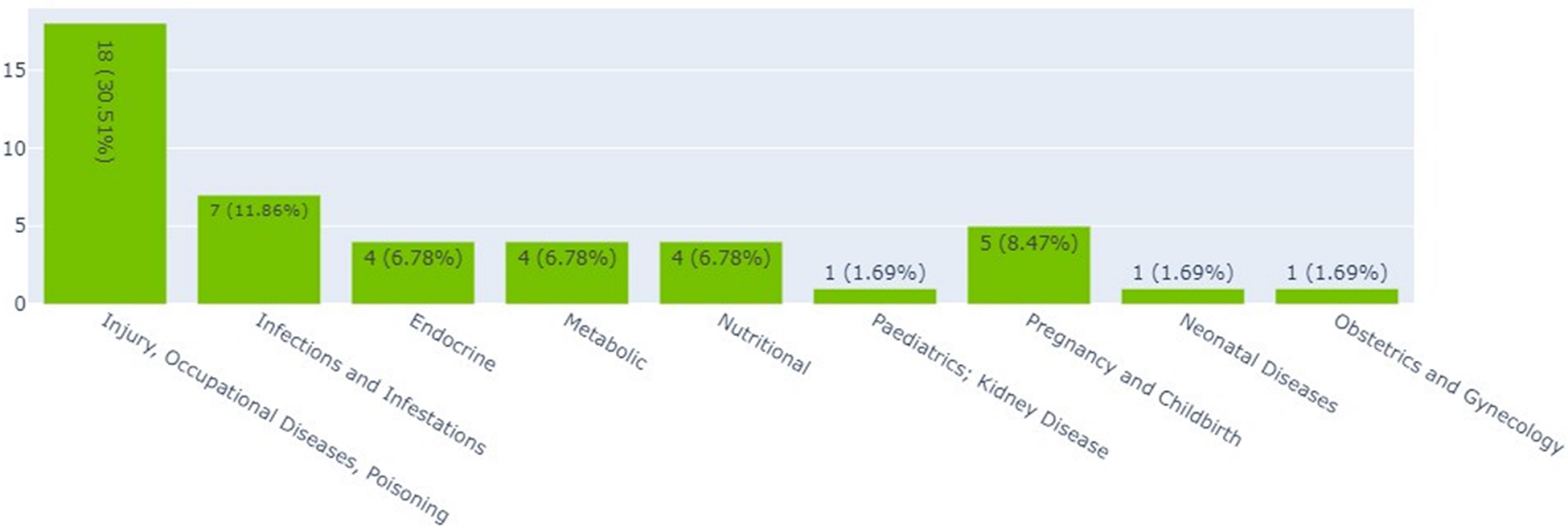
Interventional clinical trials (by thematic area) in Kenya, Ethiopia and Nigeria.

Figure 2 shows that the largest proportions of clinical trials (30.5%) were on injury, occupational diseases, and poisoning (18). The smallest proportion of clinical trials was on neonatal diseases and obstetrics and gynecology, each accounting for 1.7% (1).

#### Phases of interventional clinical trials in Kenya, Ethiopia, and Nigeria

**Figure 3:**
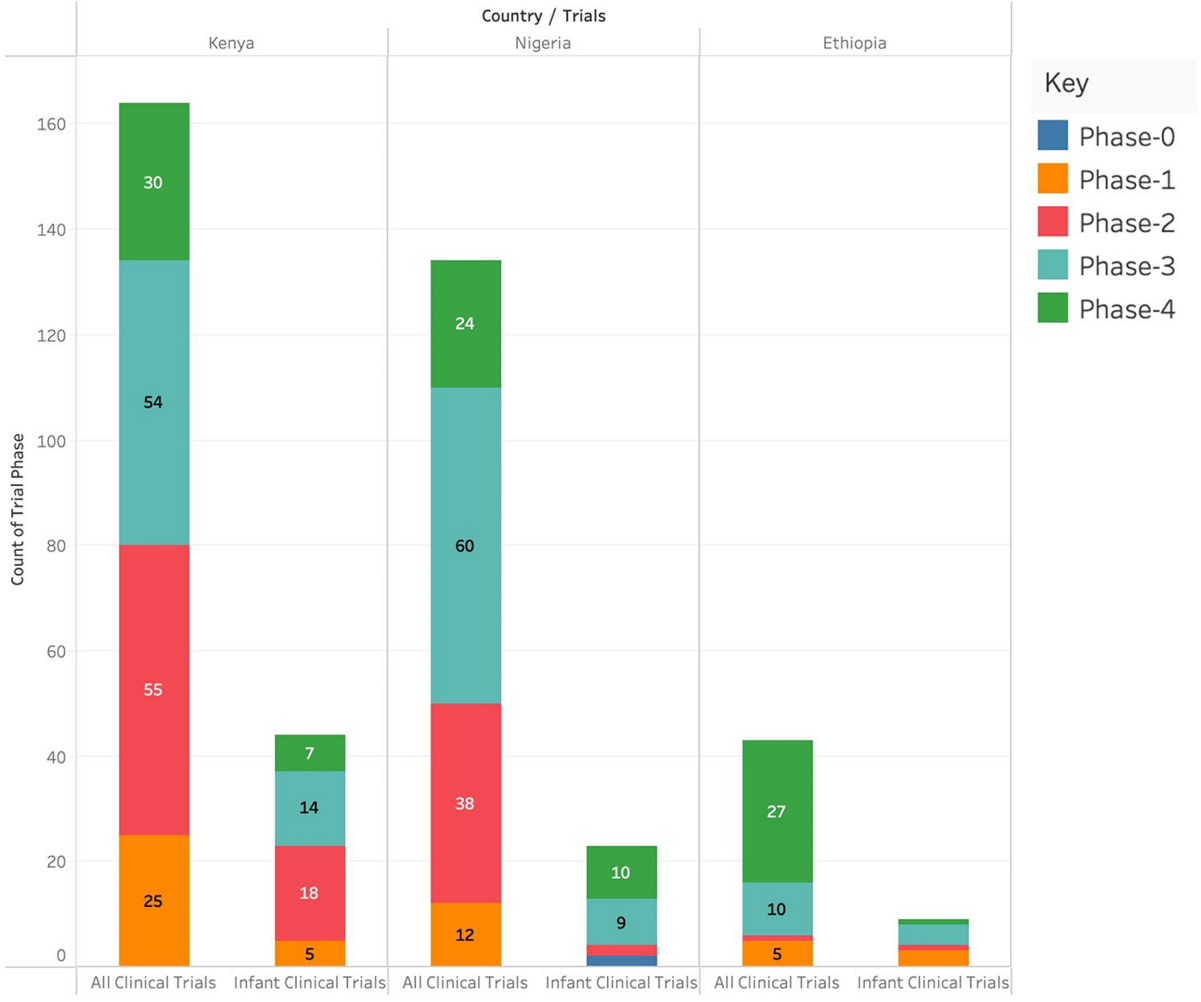
Number of different clinical studies (categorized by phases) conducted in Kenya, Ethiopia and Nigeria.

Kenya had the highest number of clinical trials in Phase I (*n* = 17), Phase II (*n* = 34), and Phase IV (*n* = 20) and Nigeria had the highest number of clinical trials in Phase 3 (*n* = 44). Ethiopia had the lowest number of clinical trials in any of the phases. Kenya had the highest number of infant clinical trials in Phase I (*n* = 5) and Phase II (*n* = 8) while Nigeria had the highest number of infant clinical trials in Phase IV (*n* = 5). There were no infant clinical trials in phase III in any of the three countries as shown in Figure 3.

#### Maximum recruitment ages for clinical trials in Kenya, Ethiopia, and Nigeria

All study subjects in all studies that were conducted in Nigeria from 2015 to 2024 were less than 20 years old. All clinical trials in Kenya in 2018 were conducted on study participants who were more than 45 years of age.

#### Number of infant clinical trials in Kenya, Ethiopia, and Nigeria

Nigeria had the highest proportion (42.9%, *n* = 9) of infant clinical trials and Ethiopia had the lowest proportion (23.8%, *n* = 5).

#### Infant clinical trials in Kenya, Ethiopia, and Nigeria by thematic area

**Figure 4:**
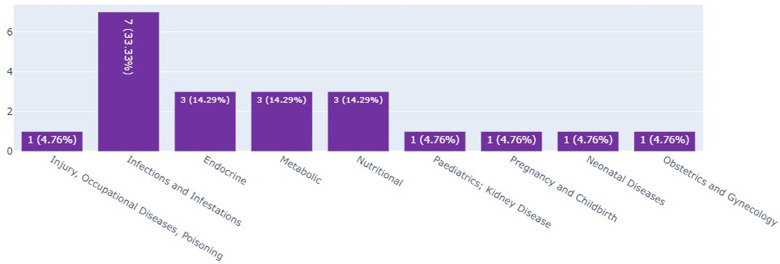
Total number of infant clinical trials in Kenya, Ethiopia and Nigeria by thematic area.

The scoping review and the landscape analysis established that most of the infant clinical trials in Kenya and Ethiopia were on infections and infectious pediatrics; these accounted for 33.3% (*n* = 7) of the total number of infant clinical trials as seen in Figure 4.

### Project data extraction from institutions carrying out interventional infant clinical trials

Data was extracted from 28 reports (10 in Kenya and Nigeria each and 8 in Ethiopia). These were the reports that were developed from the previous engagement with institutions carrying out clinical trials in the three countries and the reports had not been published. However, some of the data was incomplete. Table 2 shows the reports' distribution by country.

**Table 2:** Distribution of reports per country.

Country	Relevant reports
Kenya	10
Ethiopia	8
Nigeria	10
Total	28

#### Collaborating partners

Most reports showed that researchers conducting clinical trials in Africa were collaborating/had collaborated with either the national governments and/or international organizations or institutions like WHO and Indiana University. A PI in Nigeria reported that: “*The institute, in collaboration with the public health department of the hospital/college, usually conducts outreach services to nearby communities within the catchment area and has a cordial relationship with the communities. This has enabled us to establish a cordial and longstanding relationship on carrying out clinical trials with the surrounding communities and the state ministry”*.

#### Challenges of conducting clinical trials on infants

Researchers highlighted some challenges they have gone through while carrying out infant clinical trials. One such challenge is a lack of infrastructure that supports such trials. A PI in Ethiopia reported that: “*We have the basic infrastructure but considering infants, the ICU, and other inpatient facilities are lacking”*.

Another challenge highlighted was the lack of financial resources to carry out these types of clinical trials as stated by a PI in Ethiopia: “*We have ample experience in community-based randomized controlled trials. Although we are looking ahead to a clinical trial our main constraint is a lack of resources”*.

The link between financial resources, inflation and policies was also brought up by two researchers. A PI in Nigeria reported that: “*There are normally drastic fiscal policies and inflation in the country making the clinical trial a bit challenging. The initial budget and funding for the trial might not be sufficient for the trial at the implementation phase due to inflation and financial policies”*.

A PI in Ethiopia reported various challenges in both human resources and financial resources: “*We have inadequate access to funds and grants for conduct of research, we have inadequate incentives for research participants and the community beyond receiving the intervention, loss of some experienced personnel due to outmigration, and there is also insufficient training for co-investigators”*.

Another PI in Ethiopia pointed out that there was a lack of policy documents in their institutions/countries to carry out infant clinical trials: “*The challenge I have come through is a lack of policy documents for conducting Infant trials”*.

Researchers reported that they have experienced challenges in recruiting children in clinical trials, especially when the disease prevalence is too low, and parents refuse co-consent and assent. A Nigerian PI reported that: “*Recruitment could be a challenge especially if the prevalence of the condition of interest is not very high in the study location. Sometimes information on the burden of childhood disorders available in the public domain does not really represent the true situation in those areas. This could affect the duration of the study and might necessitate an amendment of the study protocol for change in the study location”*.

Some researchers reported that it was quite challenging to carry out both anthropometric and clinical assessments with high levels of accuracy and precision and to collect specimens from infants without causing hemodynamic disturbance in the child. A PI in Nigeria reported that: “*Anthropometric measurements such as length and mid-upper arm circumference and sample collection could be challenging in infants. Getting to position the infant properly to take that measurement is quite challenging”*.

A PI in Nigeria reported that the study participants failed to follow the study protocols as advised, especially when the trial is testing the effectiveness of products: “*Poor adherence to study protocol and loss to follow-up. Some caregivers fail to use the investigational product as instructed—especially those involved in effectiveness studies”*.

There was a reported challenge in identifying both adverse events and severe adverse events that either need hospitalization or not. This challenge arises when the study participants are not under the direct supervision of the investigators for effective studies. A Nigerian PI reported that: “*Adverse events in clinical trials can be clinical or laboratory. For effectiveness studies wherein participants are not strictly under the supervision of the investigators, adverse events might not be reported early as the caregivers might not be very conversant with their clinical manifestation while others can only be identified by laboratory investigations”*.

Even though there was a challenge in early detection, one researcher reported that their study participants often refused to be hospitalized during the occurrence of adverse events requiring hospitalization. A PI in Nigeria stated that: “*Some caregivers decline hospitalization for children that get severely ill in the course of the trial. Their refusal is usually hinged on the fact they are the ‘breadwinners’ of their families. Family income will be adversely affected when they stay on admission.”*

#### Capacity to carry out clinical trials

A PI in Ethiopia reported that their institution has laboratories to effectively carry out infant clinical trials: “*Our institution has three laboratories with seven sub-sections (Anthropometric Measurement, Biochemical Analysis, Nutrient Analysis, Clinical Assessment, Food Chemistry, Food Microbiology, Food Product Development, and Sensory Evaluation Laboratory)”*.

There are also adequate human resources to effectively carry out infant clinical trials. The Ph.D. students assist in conducting the trials. A PI in Ethiopia reported that: “*We have an adequate number of Ph.D. students both in human nutrition and food sciences for rigor evidence”*.

In addition to human resources capacity, a PI in Ethiopia reported that they have varied capacities which include capacity in human resources, administrative management, structural capacity, and grant management facilities to effectively carry out infant clinical trials *“The institution has the requisite human, administrative capacity, structural capacity, and grant management facilities to effectively conduct infant clinical trials”*.

Another PI in Ethiopia reported that they have the capacity to collaborate with both African and non-African institutions in fundraising efforts: “*Recently our team secured a grant to conduct research from a United States-based funder. I was also appointed a Research fellow at the University of KwaZulu-Natal Durban South Africa, I also secured some grants in my other areas of research interests and held a PhD”*.

#### Areas for potential collaboration

The researchers highlighted specific areas of potential collaboration which include traditional medicine, clinical development, aflatoxin exposure, maternal health, adult non-communicable diseases, adult communicable diseases, infant nutrition, hypoxic-ischemic encephalopathy, and alternatives to head cooling in LMIC, community kangaroo mother care, among others. A PI in Ethiopia reported that: “*we would wish capacity strengthen the local health innovation and clinical development of traditional medicines for the improvement of patient care in Ethiopia”*.

While most researchers were clear on specific areas of collaboration, some did not have specific areas as evidenced by a PI in Kenya who reported that: *“We would want a collaboration in community-based clinical randomized trials in any field of pediatrics and child health as the institution has established study sites”*.

#### Opportunities for joint research projects and capacity-building initiatives

Some researchers reported that some opportunities for capacity building include training in research ethics conduct, grant writing and administration, good clinical practice, data management and statistics, trial monitoring, good laboratory practice, biosafety, and identification and response to adverse events. An opportunity for joint research projects can start with collaborative grant proposal writing. A PI in Nigeria reported that: *“We need training for principals and co-investigators on the ethical conduct of research. We also need training on the grant application, administration, and writing of winning proposals”*.

#### Results and impact of clinical trials

Some researchers reported that the results of some of the community-based clinical trials were used to justify the generation of further evidence in addition to supporting the development of various interventions. A PI in Ethiopia reported that: *“The results of some of the community-based clinical trials were used to call for further evidence as input for the formulation of large-scale nutrition interventions and optimizing the nutrition impact. The reports were communicated to all nutrition actors. Furthermore, this implementation research encouraged the continuing evolution of the body of evidence available to inform nutrition policy in Ethiopia and similar settings”*.

Some researchers from Nigeria stated how the results from the clinical trials had an impact by being utilized by the federal government in their country. A Nigerian PI reported that: “*The data on Pediatric bacterial meningitis is being used by the Federal government to monitor the impact of Pneumococcal and Hib vaccination in children and this study is also jointly carried out by the institution and the Federal Government”*.

Another PI in Nigeria reported that their clinical trial has had an impact on policies in their country by helping in improving patient care management of resources and advancing research in diverse areas of clinical decision-making, that is: *“The Maggot therapy which is now used to treat diabetic foot has saved a lot of patients from amputations and saved a lot of resources and changed policy in terms of management of patients. Without clinical trials, these cannot be achieved in our institution”*.

## Discussion

This study used desk reviews to identify interventional clinical trials that have been conducted/are being conducted in Kenya, Ethiopia, and Nigeria between 2015 to May 2023. The project data extraction aspect identified some thematic areas, a list of collaborating partners, challenges in contacting clinical trials, capacities of African institutions to conduct clinical trials, opportunities for joint research projects and capacity-building initiatives, areas for potential capacity building, and the impact of some of the clinical trials.

This study identified 24 institutions in Ethiopia carrying out interventional clinical trials and also identified 28 studies on clinical trials. The study findings are in line with an earlier study which showed that only a limited number of clinical trials from Ethiopia (1.5%, *n* = 59 clinical trials) are registered with trial registries (3). These broadly reflect the inequality of clinical research and service capacity in Africa and the world at large. In Kenya, this study identified 17 institutions carrying out clinical trials and 113 clinical trials. This is contrary to an analysis done by Boston University which showed that at any given time, there were about 200 clinical trials being conducted in Kenya (19). It should be noted that the Boston University report highlighted that these 200 studies were both observational and interventional, while this study focuses on interventional studies. Additionally, information on whether a study was only registered or had made progress in various phases was not available. In Nigeria, this study identified 32 institutions carrying out clinical trials and 97 clinical trials. These numbers are very low compared to the numbers reported by WHO in 2022 (20). This study's findings for both Kenya, Ethiopia, and Nigeria are fewer than the number of clinical trials listed in the WHO International Clinical Trials Registry Platform (ICTRP) reported between 2015 and 2022. The ICTRP showed that, during this period, Kenya had 767 clinical trials, Nigeria had 775 and Ethiopia had 350 (20). However, the ICTRP report comprised both interventional, observational, and unknown trials while our report comprised only interventional trials. Additionally, ICTRP reports the clinical trials at the point of registration while our study aimed at obtaining information on whether the clinical trials were carried out and challenges in conducting the clinical trials.

This study showed that the number of interventional clinical trials in Kenya increased steadily from 2015 to 2019 before sharply reducing from 2019 to 2020 and then increasing again in 2021. This study's findings align with the 2022 WHO report which showed that the number of clinical trials in Kenya reduced sharply in 2019 and later started increasing steadily from 2021. This study found that the number of clinical trials in Nigeria reduced in 2021, which is reflected in the 2022 WHO findings. Our findings showed that the number of clinical trials in Ethiopia increased steadily from 2021, which is contrary to the 2022 WHO report that shows that the number of clinical trials in Ethiopia decreased steadily from 2021 (20). This study identified five interventional infant clinical trials in Ethiopia, 28 in Kenya, and 11 in Nigeria. Our findings are similar to those of the 2022 WHO data which reported 7 interventional infant clinical trials in Ethiopia, 23 in Kenya, and 6 in Nigeria (20).

Results from this study showed that the highest number of clinical trials in Kenya, Ethiopia, and Nigeria was in the area of injury, occupational diseases, and poisoning. Infections and infestations came second. According to WHO, most of the studies registered in Kenya (*n* = 34), Ethiopia (*n* = 6), and Nigeria (*n* = 16) between 2015 and 2022 were on infections and infestations. On infant clinical trials, this study established that there were more of these trials in Kenya (*n* = 3) and Ethiopia (*n* = 3) in the area of infections and infestations. For Kenya and Ethiopia, this study's findings are in line with the WHO 2022 report which showed that the highest numbers of infant clinical trials for both Kenya and Ethiopia were on infections (*n* = 11) and infestations (*n* = 3). This study's findings differ from the 2022 WHO report that highlighted that most infant clinical trials in Nigeria were in the area of maternal hemorrhage (*n* = 3) (20).

The landscape review showed that Kenya had the highest number of clinical trials in Phase I, Phase II, and Phase IV while Nigeria had the highest number of clinical trials in Phase III. Ethiopia had the lowest number of clinical trials in all phases. These findings match the 2022 WHO report which showed that Kenya had three Phase I clinical trials, 11 Phase II, 10 Phase III, and 2 in Phase IV. The report showed that Nigeria had two, five, three, and one clinical trials documented in Phases I, II, III, and IV, respectively while Ethiopia had three clinical trials in Phase III and one clinical trial in Phase IV (20). There are more clinical trials registered on the WHO platform than the numbers reported in each phase. This could be an indication that some clinical trials are registered and fail to kick off/are not tracked. We recommend that concerned stakeholders should come up with a tracking system that not only captures the clinical trial at registration but also until completion.

The analysis of the project data from this study showed that there was a lot of collaboration among the institutions carrying out clinical trials and that this has contributed to their success. These findings are supported by previous studies which found that most academic authors and researchers found collaborations with funders and other institutions beneficial (21). It is recommended, therefore, that deliberate efforts be made to foster collaboration between institutions carrying out clinical trials and funders and other institutions both locally and internationally.

This study established major challenges in carrying out clinical trials, that is, lack of finances, infrastructure, and human resources as well as challenges in the study participants' recruitment and in clinical/anthropometric assessments for the infants. Previous studies have also shown that the main challenges in carrying out clinical trials include: a lack of research environment, financial and human capacity, ethical and regulatory system obstacles, and operational barriers (17). This study established that human resources, infrastructure, and good grant management and administration are key to effectively carrying out clinical trials. These findings are aligned with the implementation strategy of the European and Developing Countries Clinical Trials Partnership (EDCTP2) programme which has worked towards developing and strengthening human capital and institutional capacities in SSA to undertake high-quality clinical research (22). While this study has found that clinical trials have had an impact on policies and practices, an earlier study showed that clinical trials hardly translate to outputs and impacts that can lead to policy changes (23).

There were a few methodological limitations in this study. The study only included clinical trials reported in the English language and as such clinical trials in other languages from the three countries were not captured. Further, the desk reviews could not establish non-African researchers based in Africa and conducting clinical trials in Africa. Additionally, this study may be subject to bias (recall, social desirability, and selection biases), particularly on self-reported data from stakeholders involved in clinical trials in these countries. We also note that each country may have its own unique regulatory framework for clinical trials, which could differ from international standards, and this could make it challenging to compare and synthesize the findings across the three countries.

Data on clinical trials can effectively be managed through tracking clinical trials from registration to the closure of the clinical trials. It is also ideal to conduct a post-evaluation of the clinical trials to document resulting impact or lack thereof. Collaboration with the government, funders, local institutions and international institutions is key to the success of clinical trials. Solutions to challenges can always be implemented to ensure that clinical trials are completed. A potential area of future research could be exploring the reasons why most clinical trials are registered and don't kick off/ are not monitored after registration. The findings of this study contribute to new evidence to the currently small but growing evidence on the mapping of clinical trials in Africa. This study informs the need for interventions to track clinical trials after registration, capacity-strengthening interventions, and collaborations with different stakeholders.

## Conclusion

This study identified clinical trials and infant clinical trials in Kenya, Ethiopia and Nigeria in order to identify opportunities for conducting clinical trials in these settings as well as to highlight issues related to infrastructure, regulatory frameworks, funding, collaboration and capacity-building. The findings of this study inform policy and practice in these countries and help improve the quality and quantity of clinical research in these settings through tracking efforts to track the progress of clinical trials or/and capacity strengthening initiatives. The study could also contribute to the global body of knowledge on clinical research in LMIC and help advance the goal of ensuring equitable access to safe and effective health interventions for all.

## Data Availability

The original contributions presented in the study are included in the article/Supplementary Material. Further inquiries can be directed to the corresponding author.
